# A Persuasive mHealth Behavioral Change Intervention for Promoting Physical Activity in the Workplace: Feasibility Randomized Controlled Trial

**DOI:** 10.2196/15083

**Published:** 2020-05-04

**Authors:** Md Sanaul Haque, Maarit Kangas, Timo Jämsä

**Affiliations:** 1 Research Unit of Medical Imaging Physics and Technology Faculty of Medicine University of Oulu Oulu Finland; 2 Medical Research Center Oulu Oulu University Hospital and University of Oulu Oulu Finland; 3 Department of Diagnostic Radiology Oulu University Hospital Oulu Finland

**Keywords:** mHealth behavioral change intervention, persuasive app, UCD, game elements, physical activity, SDT

## Abstract

**Background:**

Employees in an office setting are more likely to remain physically inactive. Physical inactivity has become one of the major barriers to overcoming the risk factors for anxiety, depression, coronary heart disease, certain cancers, and type 2 diabetes. Currently, there is a gap in mobile health (mHealth) apps to promote physical activity (PA) for workers in the workplace. Studies on behavior change theories have concluded that health apps generally lack the use of theoretical constructs.

**Objective:**

The objective of this study was to study the feasibility of a persuasive app aimed at encouraging PA among employees and to understand the motivational aspects behind the implementation of mHealth apps among office workers.

**Methods:**

A 4-week study using a mixed methods (quantitative and qualitative) design was conducted with office-based employees in cities in 4 countries: Oulu, Finland; Carlow, Ireland; London, United Kingdom; and Dhaka, Bangladesh. Of the 220 invited participants (experimental group, n=115; control group, n=105), 84 participated (experimental group, n=56; control group, n=28), consisting of working-age volunteers working in an office setting. Participants used 2 different interventions: The experimental group used an mHealth app for PA motivation, and the control group used a paper diary. The purpose was to motivate employees to engage in healthier behavior regarding the promotion of PA in the workplace. A user-centered design process was followed to design, develop, and evaluate the mHealth app, incorporating self-determination theory (SDT) and using game elements. The paper diary had no specific theory-driven approach, design technique, nor game elements.

**Results:**

Compliance with app usage remained relatively low, with 27 participants (experimental group, n=20; control group, n=7) completing the study. The results support the original hypothesis that the mHealth app would help increase PA (ie, promoting daily walking in the workplace) in comparison to a paper diary (*P*=.033). The mHealth app supported 2 of the basic SDT psychological needs, namely autonomy (*P*=.004) and competence (*P*=.014), but not the needs of relatedness (*P*=.535).

**Conclusions:**

The SDT-based mHealth application motivated employees to increase their PA in the workplace. However, compliance with app usage remained low. Future research should further develop the app based on user feedback and test it in a larger sample.

## Introduction

Lack of physical activity (PA) affects normal physiological processes in the human body, which may destabilize the body's energy balance, cause muscle atrophy, and diminish exercise capability [1]. Physical inactivity has become one of the major barriers to overcoming the risk factors for obesity, stroke, type 2 diabetes, and mental health issues. These risk factors can cause long-term disease that lead to death [2]. Several factors may discourage participation in PA (eg, a lack of parks, paved areas, and sports or recreation facilities), and the World Health Organization member states have set a target to reduce physical inactivity up to 10% by 2025 [3]. Despite this, some individuals remain persistently physically inactive, thus leading to a high risk of medical complications and causing significant health care expense [4,5]. The consequences may be compounded by physical inactivity in the workplace. Interventions that are designed and developed for the workplace environment are encouraged and may result in progress in PA [6].

Technology-enhanced solutions are a promising approach to motivating people and promoting PA. Mobile health (mHealth), using smartphones for health-oriented applications, has emerged as a vital tool for health-oriented behavioral change interventions [7] and to reduce health problems [8].

Persuasive health apps have been proposed as a technique to foster behavioral change [9-11]. State-of-the-art behavioral change efforts are essential for increasing PA promotion [12]. However, mHealth apps are not generally grounded in behavior change theories [13-15].

According to the self-determination theory (SDT), people can be intrinsically and extrinsically motivated to perform an action [16,17]. An intrinsic level of motivation is completed through the fulfillment of the 3 psychological needs: autonomy, competence, and relatedness. Autonomy shows a sense of having the option to measure the social environment and distributing selections that conform to carrying out a daily task [18]. Competence indicates a sense of completing the task in a social environment. Relatedness specifies the feeling of working to connect with others [18]. Hence, SDT is a promising method for overcoming the challenges of physical inactivity in the work environment and a lack of social interaction among employees. Workers can be motivated intrinsically (ie, they feel gratified in performing their daily walking routine). Then, they are extrinsically more motivated to complete their PA task, since they want to finish the job (eg, they can track their progress by scoring points and earning badges based on a leaderboard during their daily walk). However, employees who are amotivated may not demonstrate an awareness to perform any level of their daily PA task. Thus, intervention strategies that are purported to satisfy the 3 needs of SDT might encourage positive behavioral change [19].

Gamification is the use of game elements in non-gaming contexts [20] and motivating individuals by making their experience more fun and playful [21]. Human behavior is motivated by extrinsic aspects such as incentives or rewards that have been utilized to encourage motivation among employees [22,23]. Virtual points and badges are ways of representing rewards [24]. Furthermore, competition is a persuasive technique derived from the Theory of Competition [25], referring to “the act of seeking or endeavoring to gain what another is endeavoring to gain at the same time” [26]. A leaderboard is a way to represent competition in which users’ activities are demonstrated [27]. The implementation of rewards is a practical way to foster users’ behaviors in non-gaming contexts [28]. In PA research, points, badges [29-35], and leaderboards [29-31,34,35] (PBL) can persuade individuals to complete a specific activity.

As a potential solution to increase PA in the workplace, this study designed and developed a persuasive mHealth app called iGO that incorporates SDT. SDT was selected for its acknowledgment in PA research [36] and its capability to support an individual’s behavior by offering reinforcement of the 3 basic psychological needs: autonomy, competence, and relatedness. The design followed the user-centered design (UCD) process [37]. The iGO mHealth app allowed users to set goals for PA after breakfast and lunch sessions and enabled them to track their walking performance. The purpose was to motivate employees to increase their daily PA and social interaction among colleagues and others. This study aimed to answer the following research questions:

1. What is the feasibility that the persuasive iGO app will motivate employees to increase their daily walking in the workplace?
2. What is the employees’ view of the persuasive iGO app regarding the needs of autonomy, competence, and relatedness for promoting daily walking?

To answer the research questions, we evaluated the iGO mHealth app during a 4-week study with a mixed methods (quantitative and qualitative) design. We hypothesized that iGO would motivate employees to increase their daily walking and increase their autonomy (confidence level and ability to choose regular walking to reach their goal), competence, and relatedness.

## Methods

### Study Design

An experimental study was conducted with a group of office-based employees for 4 weeks. Participants were randomly assigned to 1 of 2 groups. The experimental group used the iGO mHealth app, and the control group used a paper diary on weekdays for 4 consecutive weeks.

### App Design

iGO was iteratively developed in previous studies, and the app design has been presented elsewhere [38,39]. Briefly, we designed and developed the gamified, persuasive mHealth app iGO [39] to encourage employees to walk more often and to break up long sitting periods during working hours. iGO is based on the SDT-driven system model [38]. This model is a combination of the SDT theory, game elements, and motivating outcomes (exercise, walking, and weight control). The SDT theory model of health behavioral change defined by Ryan et al [40] was adopted. The iGO app utilizes the 3 basic SDT psychological needs and aims to motivate users to increase their daily PA by increasing their levels of autonomy (ability to choose a daily walking task to reach a 10-minute goal after breakfast and lunch breaks), competence (feeling effective in their ongoing interactions with the social environment and to reach the daily walking goals), and relatedness (feeling connected with colleagues for the purpose of PA).

#### User-Centered Design (UCD)

Our proposed gamified system model was used to design and develop a low-fidelity prototype (paper prototype) of iGO applying the UCD process, consisting of 5 steps: empathize, define, ideate, prototype, and test. The details have been published elsewhere [38]. The prototype allowed users to enter their information, such as name, age, and weight. By logging into the app, the user had the option to participate in PA with others or alone [38]. Every 5 minutes of PA resulted in 1 point; therefore, a 5-minute walk was awarded 1 point, and a 10-minute walk was awarded 2 points. The users could monitor their activities on the leaderboard. The iGO app prototype [38] was tested by 5 volunteers. Based on the users’ recommendations, a high-fidelity prototype of the iGO mHealth app was designed and evaluated during a 1-week study [39] and further upgraded to a newer version of the iGO mHealth app, as presented in the following sections.

#### Working Version of the iGO mHealth App

**Figure 1:**
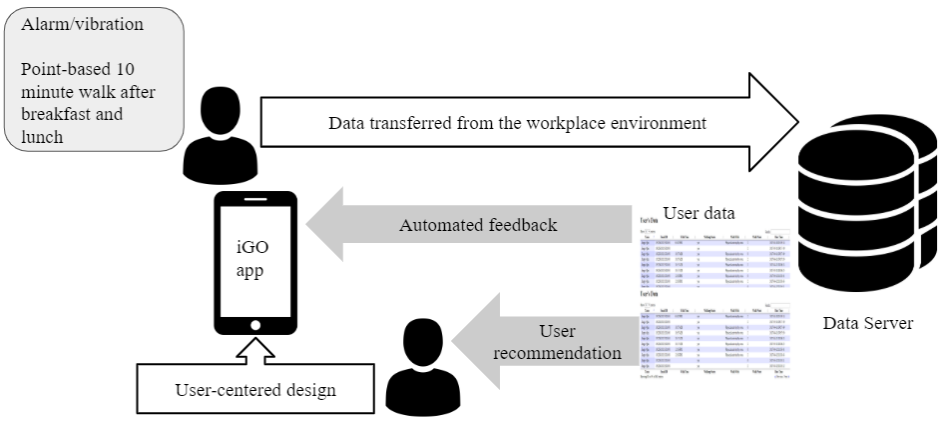
Overall concept of the mobile health app iGO, developed using the user-centered design process.

The overall concept of the iGO app is shown in Figure 1. Users' key characteristics (comprising intrinsic motivation and levels of autonomy, competence, and relatedness with regard to their PA participation) and responses were collected from the system. In addition, their daily walking activity was logged automatically by the system. Users received notifications and their progress-related rewards are displayed on the leaderboard.

**Figure 2:**
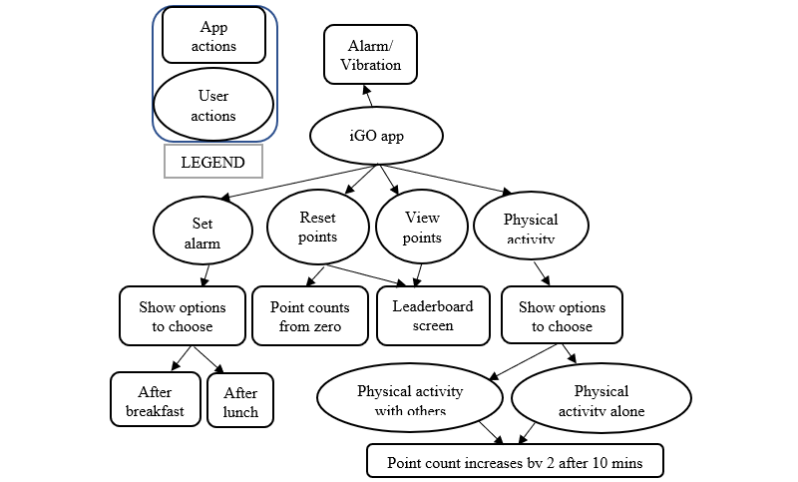
Flow within the iGO mobile health app.

The flow within the iGO mHealth app is shown in Figure 2.

The iGO app (Multimedia Appendix 1) provides a choice in the main menu to select “yes” or “no” regarding the user’s breakfast or lunch status. If the user selects “no” to indicate he or she did not have breakfast, the alarm reappears after 10 minutes and asks the user to select an option to proceed. Selecting “yes” will ask the user for a preference — either “physical activity with others” or “physical activity alone” — and the activity is timed for 10 minutes. Users have the choice to accept or skip the app function by pressing the “yes” or “no” button when they are asked whether they had breakfast or lunch. Users express their views and are able to select their choices through the app. Thus, the iGO provides autonomous support. Moreover, the autonomous choice of “physical activity alone” allows users to walk up to 10 minutes or more if they wish (their walking data are stored in the data server). We included rewards such as PBL game elements in the system to motivate participants to walk for 10 minutes and add points to meet their goal.

**Figure 3:**
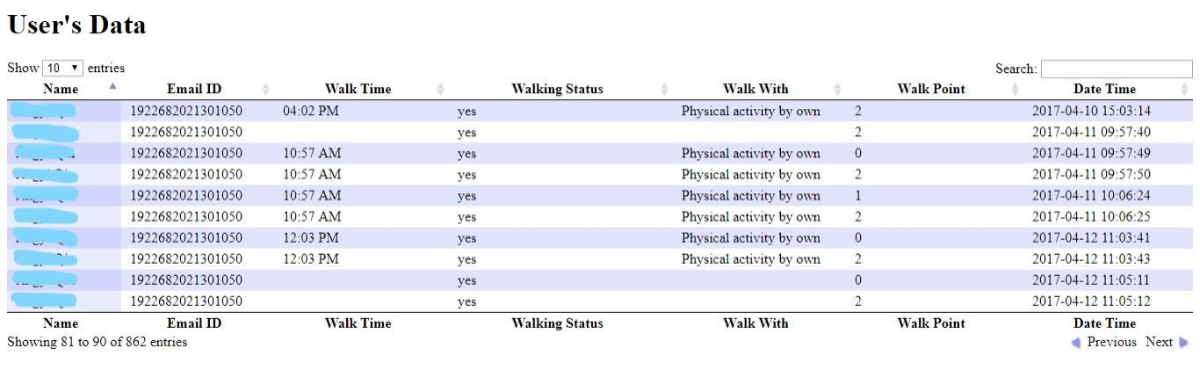
A user’s walk and step count after the lunch recess are tracked, and the data are gathered in the web server.

The accelerometer sensor in the smartphone tracks the footsteps of the users, targeting 1000 steps in 10 minutes, based on the recommendation of 3000 steps in 30 minutes [41]. Here, 500 steps are counted as 1 reward point. Reminders are sent via an alarm/vibration during breakfast and lunch. The leaderboard appears as an interactive social display when the users select “View points” from the main menu. The leaderboard shows the ranked list of users, their names, photos, and earned points. Users can customize their picture and name visibility settings when signing into their iGO account. Additional details of the user interface of the functionality pages of the iGO mHealth app have been published elsewhere [42]. Walking data are gathered on the web server (Figure 3).

An 8-week usability evaluation of the final iGO mHealth app was conducted earlier, utilizing the unified theory of acceptance and technology model. The details have been published elsewhere [42]. Briefly, the usability was evaluated based on performance expectancy, effort expectancy, social influence, facilitating conditions, behavioral intention, and use behavior. The results showed success in motivating the users to participate in PA.

### Participants

**Figure 4:**
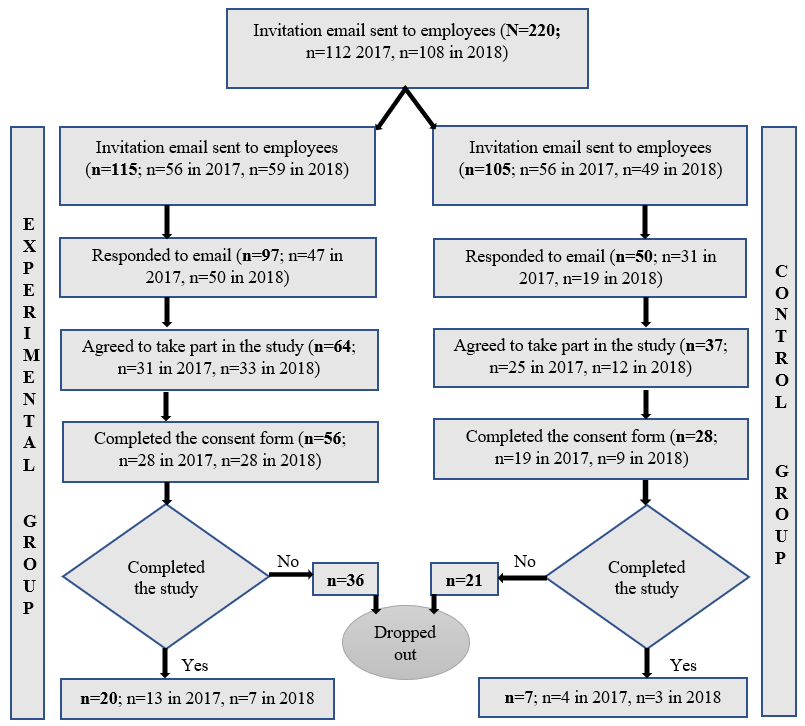
Flow chart of the study participants.

The 4-week experimental study was conducted at 4 sites in 4 countries: the city of Oulu, Northern Finland (population, ~199,000); Carlow, South Leinster, Ireland (population, ~26,000); the megacity of London, United Kingdom (population, ~8,136,000); and the megacity of Dhaka, Bangladesh (population, ~1,984,000). The 4 countries were selected due to practical reasons owing to existing collaborations. To recruit the participants, we selected 10 multinational information and communications technology companies (2 in the United Kingdom, 2 in Ireland, 3 in Finland, and 3 in Bangladesh), 1 research institute from each country (University of Oulu, Finland; Queen Mary University of London, United Kingdom; Institute of Technology, Carlow, Ireland; and Bangladesh University of Professionals, Bangladesh), and people in startup companies (Finland, United Kingdom, Ireland, Bangladesh). The list of the companies was collected, and we personally communicated with university researchers and professors. People working in the information technology sector were invited to participate, owing to the relationship between information technology skills and technology acceptance. We contacted each site by email and asked for an invitation email to be forwarded to their employees. The first author of the study contacted the people at the startup companies directly. A total of 220 people was invited. The people were randomized to the experimental (n=115) and control (n=105) groups before sending the invitations (Figure 4). Participants were randomized to the groups manually in a blindfolded randomly mixed order. They were contacted to confirm their willingness to participate in a 4-week trial. We obtained informed consent (Multimedia Appendix 2) from all participants before conducting the study. The consent form was in English in all countries. The participants were able to withdraw from the study in any phase. Based on the study design, a review by an ethical committee was not required.

The flow chart of the study participants is shown in Figure 4. The final study population consisted of 84 working-age volunteers working in an office setting who completed the consent form. The characteristics of the participants are given in Tables 1 and 2. A participant was considered to have completed the study if he or she used the app or paper diary for 4 weeks and returned the final questionnaire.

In the experimental group, 115 people were asked to participate in the 4-week trial using the iGO app. Of these participants, 56 completed the consent procedure (mean age 39 years, range 24-49 years), and 20 participants completed the study (12 men and 8 women; mean weight 72.2 kg; mean BMI 24.8 kg/m²). The reasons given for dropping out of the trial (n=36) were a lack of time, holidays, laziness, or personal issues (16/36, 44%); unwillingness to use the mHealth app because they disliked its appearance or were already using an mHealth app (6/36, 17%); did not feel a need for this type of service or were already taking care of themselves (3/36, 8%); and other reasons (7/36, 20%). A further 11% (4/36) did not give a reason for declining.

The control group (n=105) was asked to participate in the 4-week trial using a paper diary. Of these 105 people, 28 completed the consent procedure (mean age, 39 years; range 26-49 years), and 7 participants completed the study (5 men and 2 women; mean weight 71.4 kg; mean BMI 24.5 kg/m²). The reasons given for dropping out (n=21) were a lack of time, holidays, laziness, or personal issues (6/21, 28%); unwillingness to use the paper diary because they disliked it, since it was only paper, with no alarm (7/21, 35%); did not feel a need for this type of service or they were already taking care of themselves (3/21, 13%); and other reasons (6/21, 26%).

**Table 1:** Baseline characteristics of the participants.

Variable		Invited, N=220		Consented, n=84		Completed, n=27	
		E^a^, n=115	C^b^, n=105	E, n=56	C, n=28	E, n=20	C, n=7
**Gender, n (%)**							
	Female	44 (38.3)	42 (40.0)	23 (41.0)	12 (42.9)	10 (50.0)	4 (57.1)
	Male	71 (61.7)	63 (60.0)	33 (59.0)	16 (57.1)	10 (50.0)	3 (42.9)
**Work situation, n (%)**							
	University	62 (53.9)	50 (47.6)	—^c^	—	—	—
	IT^d^ industry	36 (31.3)	41 (39.0)	—	—	—	—
	Startup company	17 (14.8)	14 (13.4)	—	—	—	—
**Country, n (%)**							
	United Kingdom	32 (27.8)	30 (28.5)	18 (32.1)	9 (32.1)	6 (30.0)	2 (28.6)
	Ireland	22 (19.1)	19 (18.1)	12 (21.4)	5 (17.9)	6 (30.0)	1 (14.3)
	Finland	26 (22.6)	24 (22.9)	15 (26.8)	8 (28.6)	5 (25.0)	3 (42.9)
	Bangladesh	35 (30.4)	32 (30.5)	11 (19.6)	6 (21.4)	3 (25.0)	1 (14.3)

^a^E: experimental group.

^b^C: control group.

^c^Data were not available.

^d^IT: information technology.

**Table 2:** Baseline characteristics of the participants by country.

Variable		Invited, N=220		Consented, n=84			Completed, n=27		
		E^a^, n=115	C^b^, n=105		E, n=56	C, n=28		E, n=20	C, n=7
**United Kingdom, n (%)**									
	Female	11 (34.4)	13 (43.3)		7 (38.9)	3 (33.3)		2 (33.3)	1 (50.0)
	Male	21 (65.6)	17 (56.7)		11 (61.1)	6 (66.7)		4 (66.7)	1 (50.0)
**Ireland, n (%)**									
	Female	10 (45.5)	7 (36.8)		5 (41.7)	1 (20.0)		3 (50.0)	0 (0)
	Male	12 (54.5)	12 (63.2)		7 (58.3)	4 (80.0)		3 (50.0)	1 (100)
**Finland, n (%)**									
	Female	11 (42.3)	9 (37.5)		6 (40.0)	4 (50.0)		3 (60.0)	2 (66.7)
	Male	15 (57.7)	15 (62.5)		9 (60.0)	4 (50.0)		2 (40.0)	1 (33.3)
**Bangladesh, n (%)**									
	Female	12 (34.3)	13 (40.6)		5 (45.5)	4 (66.7)		2 (66.7)	1 (100)
	Male	23 (65.7)	19 (59.4)		6 (54.5)	2 (33.3)		1 (33.3)	0 (0)

^a^E: experimental group.

^b^C: control group.

### Paper Diary

The paper diary had a simple chart to record walking after breakfast and lunch. The diary did not incorporate SDT needs and PBL elements. Users entered their data manually. Participants were instructed to complete their walking record on the paper diary during their breakfast and lunch breaks every weekday for 4 weeks. An alarm/vibration as a reminder was not included with the paper diary (Multimedia Appendix 3).

### Questionnaire

Based on the users’ feedback in the previous study [38], a quantitative questionnaire (Multimedia Appendix 4) was initially designed, validated, and tested. The validation was conducted by 4 experts with similar research backgrounds. The questionnaire was co-designed by 6 end users living in Finland and Ireland. The questionnaire used a 7-point Likert scale, with answers ranging from “Much worse” to “Much better” (for increasing PA) and “Completely disagree” to “Completely agree” (for autonomy, competence, and relatedness). Similarly, a set of qualitative questions (Multimedia Appendix 4) was designed and tested to determine how the mHealth app or paper diary helped users improve their PA, any personal approaches used in the app or paper diary to help with their PA, any ways in which the iGO mHealth app or paper diary failed to help users or made their PA worse, and how the app could be improved.

### Interview

Semistructured 20-minute interviews [43] were conducted and audio recorded with all participants in the experimental group who completed the study. They were asked about the external contexts as well as their opinions and feelings on the usage of the iGO app. To evaluate the responses to open questions, conventional content analyses were performed by the first author. Microsoft Excel (Microsoft Corp, Redmond, WA) was used to store and organize the data collected during the interviews. Analysis was carried out in three steps: (1) the data were repeatedly read for familiarity, (2) words or phrases corresponding to the key themes were highlighted and coded, and (3) the context and frequency of theme-related sentences were recorded.

### Procedure

The iGO app was first installed on the participant’s smart device. Samsung (Seoul, South Korea) Android phones were lent to any participants who did not have a compatible phone. Participants were instructed to use the mHealth app or paper diary for 4 weeks. Participants in the experimental group were asked to use the mHealth app daily for at least 10 minutes after breakfast and 10 minutes after lunch, while walking during their break time. The participants customized the breakfast and lunch times to their preferred times. Based on the set time, the phone initiated a vibration/alarm as a reminder for participants to start walking. After walking for 10 minutes, the participants were notified by the iGO app that they completed their goal. The participants filled in the quantitative questionnaires at the end of the study.

### Statistical Analysis

The statistical tool SPSS 25.0 (IBM Corp, Armonk, NY) was used to analyze the quantitative data. Differences between the experimental group and control group were compared using *t* tests. The *P* value for each of the psychological needs of autonomy, relatedness, and competence was calculated separately. *P*<.05 was considered statistically significant.

## Results

### Quantitative Results

An overview of the quantitative questionnaire results is shown in Table 3. The answers were rated using a 7-point Likert scale, with scores of 1 to 7 corresponding to responses ranging from “Much worse” to “Much better” (for increasing physical inactivity) and to responses ranging from “Completely disagree” to “Completely agree” (for autonomy, competence, and relatedness).

**Table 3:** User ratings of whether the iGO app (experimental group) or paper diary (control group) increased physical activity, autonomy, competence, and relatedness, rated using a 7-point Likert scale.

Condition	Experimental group (n=20), mean	Control group (n=7), mean	*P* value
Increasing physical activity	6.15	4.30	.033
Autonomy	5.05	3.30	.004
Competence	4.86	2.00	.014
Relatedness	3.15	3.43	.535

The score for increasing physical activity was significantly higher for participants in the experimental group than for those in the control group (*P*=.033). Also, participants in the experimental group were significantly more likely to consider that the mHealth app increased their motivation to participate in PA alone, compared with the control group (*P*=.004).

Participants in the experimental group were significantly more likely to consider that the mHealth app increased their feeling of competence to view themselves in a social environment (ie, on the leaderboard), compared with the control group (*P*=.014). There was no difference between the experimental and control groups in their reported motivation to participate in PA with others.

Participants inputted their weight when installing the iGO app on the smartphone and transferred this information to the postexperimental questionnaires. A trend for weight loss was found when using iGO, with borderline statistical significance (pre-intervention mean weight, 72.2 kg vs post-intervention mean weight, 71.4 kg, *P*=.054).

### Open Questions

#### Autonomy

Based on the interview, most experimental group participants (14/20, 70%) were motivated, set their daily goal of 10-minute walking after breakfast and lunch, and tracked their daily walking when using the iGO app, which indicated autonomy.

I sort of liked the way how it influenced me to go for a walk and became my daily routine, liked it.

I felt like using the app has changed my habit of sitting idle in the office after breakfast/lunch.

#### Competence

Participants (13/20, 65%) felt competent when using the iGO app to view themselves in a social environment to walk daily.

[It] assisted me to interact with the phone and to walk with others

[It] became a habit, but I wanted to see more in the apps like more connection [among] people who are using it!

They felt competent using the mHealth app, which indicates fulfillment of the psychological need of competence.

#### Relatedness

Only few participants (5/20, 25%) were motivated to participate in PA (eg, walking with others) when using iGO. One participant felt connected with their colleagues and noted: “[I] walked with colleagues and friends and made me mix with others while walking; a social platform.” Perhaps iGO allowed the participant to walk daily with colleagues from the same office and track their progress from the data server. In this way, they might feel connected with colleagues. However, one participant stated: “I cannot feel any betterment to walk with others.” The sense of being connected to others using the gamified prototype is not valid in this case. Therefore, we cannot agree that the last psychological need, relatedness, was fulfilled.

### Game Elements

One participant ranked in the leaderboard reported: “I liked the way … it influenced me to go … walking and [it] became my daily routine, liked it.”

However, the social, fun part of the game elements was not apparent among the participants who reported:

Maybe add a way to create events to [attend].

Should add more socializing features.

Maybe more social fun activities options.

Participants wanted to see different points for the 2 walking conditions. One participant mentioned, “Some difference in the points for walking alone and walking with others.”

### Other Comments

The log data showed that some regular users did not upload their name and photo so they were visible on the leaderboard, even though users preferred a leaderboard that included their details when prototyping the iGO app. Within the control group, most participants suggested that some sort of reminder should be added in the paper diary.

I wish there were a kind of alarm type feature or image that [could] draw … attention towards the paper.

[A] paper diary can be easily ignored in the busy schedule, so it should be more attractive.

Of the participants who completed the study using the iGO app, 8 (8/20, 40%) participants used a provisioned Samsung phone, whereas the remaining participants used their own smartphones. The log data showed that those who used their own phone had more points and ranked higher on the leaderboard. Moreover, the data from the participants who completed the study in Ireland showed that those who ranked highly on the leaderboard (4/6, 66%) were colleagues from the same offices and building.

## Discussion

### Principal Findings

This study presents the feasibility of an mHealth app designed for promoting walking in the workplace during breaks. Based on the examination, the iGO app helped to overcome physical inactivity by increasing walking. The data supported compliance with 2 of the basic psychological needs, namely autonomy and competence, but not for the needs of relatedness. However, compliance in using the app remained low.

Satisfaction with the SDT basic needs for autonomy, competence, and relatedness is essential for establishing intrinsically motivated and sustained PA behavior [44]. In this study, most participants were motivated and set their daily goal of 10-minute walking after breakfast and lunch. Participants felt competent using iGO to view themselves in a social environment to walk daily.

Discontinuation is a central problem in technology-enhanced intervention studies [45], and participant age, gender, education level, and employment status can influence the risk of dropping out during the study [46]. In this study, the number of consented participants who did not continue the study was comparatively high. Participants were busy in their work life. They were young developers or programmers working in technology-based industries. This may indicate that the reasons for discontinuation were their young age and employment status.

Studies have shown that interventions targeting PA promotion can be designed to focus on setting-specific issues that are open to change within demographic settings differentiated by gender, age, social disadvantage, and geographic location [47]. Our users were from 4 different countries. Apart from those in the United Kingdom and Ireland who were native English speakers, participants from Finland and Bangladesh had their own native language (Finnish and Bengali, respectively). However, they all spoke English due to their international working environment. Thus, participants were not affected by the language factor when the app was in English. Instead, cultural differences may have a significant effect on the use of behavior change apps and need to be studied in more detail in future.

On the other hand, weather conditions may affect outdoor PA, as demonstrated for older adults [48]. The United Kingdom, Ireland, and Finland have varying day lengths and seasonal outdoor temperatures, in contrast to Bangladesh. Extremely cold temperatures and slippery conditions may reduce participation in outdoor activities during the winter period (eg, in Finland) but may not affect indoor behaviors. Similarly, extreme heat levels have a negative impact on human health and productivity [49], which may have limited walking outside of the office buildings in Bangladesh. The office interiors were well-equipped with air conditioning systems but may have had comparatively limited space to even walk in the office corridors. However, because the mHealth app was designed to encourage workers to walk during their breakfast or break, which they were able to do in the office area, they were not much affected by weather factors.

We did not examine the users’ recommendations, age, and gender based on the specific geographic location but averaged all users’ preferences from the 4 different countries when designing the app. The SDT needs of relatedness were absent in this study (ie, the participants did not connect with others while walking). Previous research has suggested that being connected to more people may help with PA promotion [50,51] and users tend to do more PA if they are socially connected with others for the same purpose. In this study, the environment appeared to not fully support regular PA by the participants, perhaps due to the lack of actual daily interaction with colleagues. The low levels of relatedness may be explained by the fact that few colleagues were using the iGO app at the same time. The social features of an mHealth tool have been shown to initiate positive changes in social interaction among colleagues and signing up for the tool [52]. In this study, the leaderboard included participants from the 4 countries. However, this may have demotivated participants, since it may be more motivating to compete against participants that people know personally.

Cognitive, emotional, and social benefits are credited to gamification [53]. Building positive social relationships and fostering a sense of integration are the core social benefits noted for gamification [54]. In the present study, the game elements may not have provided enough social benefits for the users. Additional features could have been added for scoring. For instance, walking with others might have scored additional points. Competition allows users to identify their situation and compare their activities to others [55]. Although a leaderboard is a way to represent competition based on users’ activity [27], competition was not noticed among the users in this study. Participants who focused more on PA than points may have earned more points than those who focused on earning points. To facilitate PA promotion, strengthening of motivation and changes in self-awareness are two essential mechanisms [56]. This suggests that participants may have been motivated intrinsically by using the mHealth app and that their self-awareness of PA in the workplace was increased through the use of the mHealth app.

Using mobile reminders is a conventional approach in health research [57-59]. The use of digital triggers (eg, alarm or vibration) can be automated in smartphones so that the users recognize the meaning of the alert. Triggers such as an adaptive control mechanism are framed to meet the needs and goals for short-term actions and longer-term behavioral change [60]. The reminders programmed in our mHealth app helped the users react for a near-term response (eg, a reminder to walk). Researchers have highlighted that mHealth solutions are still methodological and need to resolve privacy issues [61], such as security features that include secure encryption and two-factor authentication [62]. Some participants might have chosen to omit their name or photo on the leaderboard because of their preference to not disclose their daily PA-based track record (eg, total minutes of walking, earned points) to others.

Compliance with the paper diary was comparatively low, compared with that of the mHealth app. Participants forgot to complete the paper diary, it was less attractive, there was no alarm system, and it was difficult to record walking time on paper.

Walking can be beneficial for weight loss [63] and other health outcomes [64,65]. mHealth interventions with theory-based podcasts, social support [66], encouraged self-tracking [66,67], and health coaching [68] have been shown to result in weight loss. During this 4-week study, there was a trend for weight reduction among the experimental group. It is unknown whether the mHealth app influenced weight loss, since the follow-up time was relatively short, the sample size was limited, and only self-reported weight data were used.

### Limitations and Future Study

This study has some limitations. First, the final sample size was relatively small. Larger-scale studies are needed to confirm the findings. Many consented participants, who typically were young programmers or developers, discontinued the 4-week study. A next-generation solution should be designed based on the feedback received, in order to increase compliance. Second, participants were randomized before they received their invitations. This may have influenced the difference in the number of participants between the groups.

Another limitation of the study is that the weight was self-reported and collected only at the beginning and end of the study. However, this study targeted an increase in PA, not weight loss, which may reduce the possibility for bias.

The iGO app was still a prototype with somewhat limited graphical design and user interface. Some participants may have failed to connect with others due to the contemporary design of the mHealth app. PA measured by a smartphone can underestimate the number of steps, as people may leave their phone on their office desks. Fewer measured minutes of walking may result in fewer points and therefore an inaccurate leaderboard, which could demotivate participants to follow ranks and points on the leaderboard. Furthermore, the accelerometer sensor was not installed in some smartphones, and users were not able to track their steps. Hence, the actual walking data may be different from the data in the web server. The measured increase in PA was obtained from questionnaire data. We used the accelerometer-based step count to calculate the rewards. However, we did not use the objective PA data for other purposes in this study.

More features and options could be added, such as socializing functions and social, fun activities allowing users to interact with others [69,70]. Adding a gamified social networking platform [71,72] with more user-friendly social features, such as a more specific social game element, might allow users to interact with others via chat messages in the mHealth app. Points could be awarded and redeemed in schemes such as the Tesco Clubcard [73,74] and Carrots Rewards App [75]. The iGO app could have options for users to share each other’s points and perhaps exchange points for social voucher cards.

### Conclusions

In the research for this paper, we conducted a feasibility study on the mHealth persuasive app for promoting PA in the workplace. This mHealth app was developed by incorporating the SDT theory and applying game design elements. The design of the app followed the UCD process. A 4-week study was conducted with a group of office workers. The mHealth app supported users to increase their PA at the workplace, when compared with a paper diary. The iGO app fulfilled the SDT basic needs of autonomy and competence, but not relatedness (ie, it did not support participants in feeling connected with others). This study demonstrates how even a simple mHealth app can help employees increase their PA. The design of the app appeared to be a successful approach that is viable for future persuasive apps. Future research should aim to develop the app further based on users’ feedback and test it on a larger scale, enabling the critical components within the mHealth intervention to be studied.

## Appendix

Multimedia Appendix 1 User interfaces of the iGO app.

Multimedia Appendix 2 Consent form.

Multimedia Appendix 3 Paper diary.

Multimedia Appendix 4 Questionnaire.

Multimedia Appendix 5 CONSORT-EHEALTH Checklist V1.6.

## Abbreviations

mHealth: mobile health
PA: physical activity
PBL: points, badges, and leaderboards
SDT: self-determination theory
UCD: user-centered design
