# Supplementary figures and images for: A Persuasive mHealth Behavioral Change Intervention for Promoting Physical Activity in the Workplace: Feasibility Randomized Controlled Trial
Source: JMIR Form Res. 2020 May 4;4(5):e15083. doi: 10.2196/15083 (PMC7235808; doi:10.2196/15083)

Appendix 1 – User Interfaces for the iGO App.


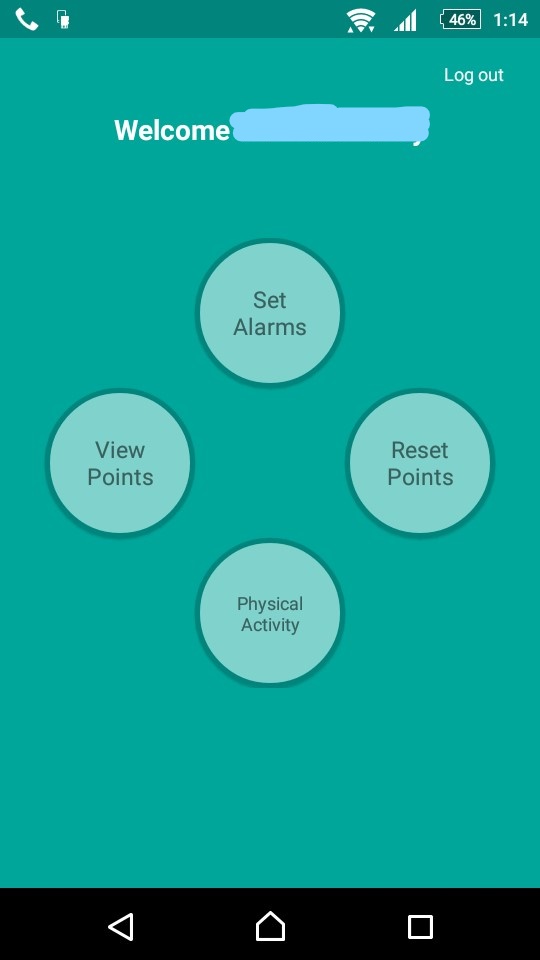


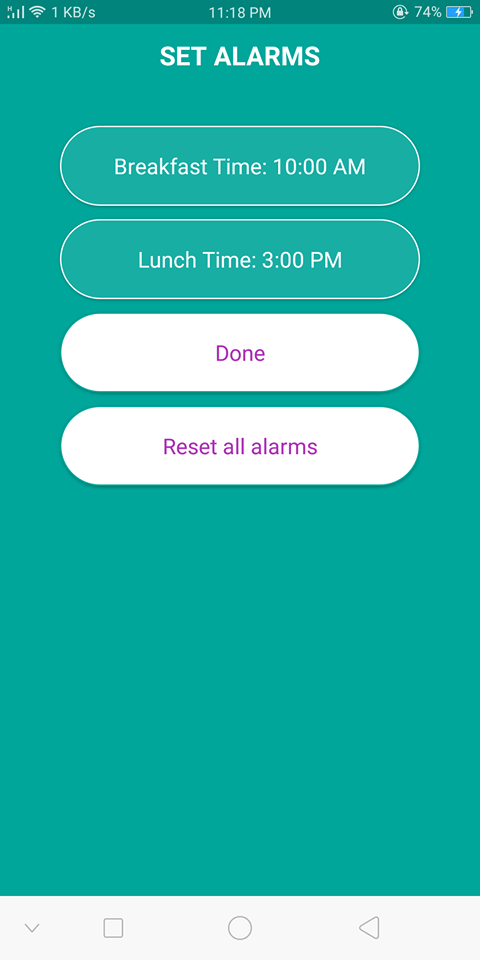


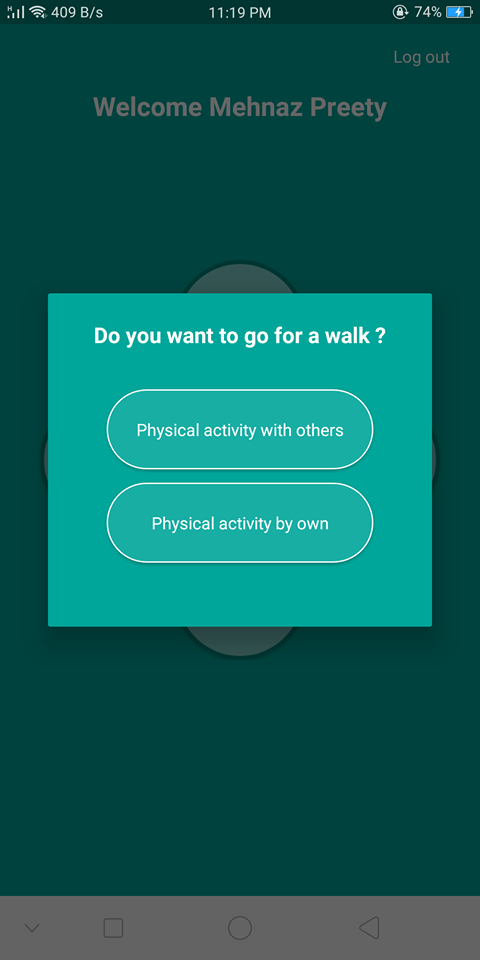


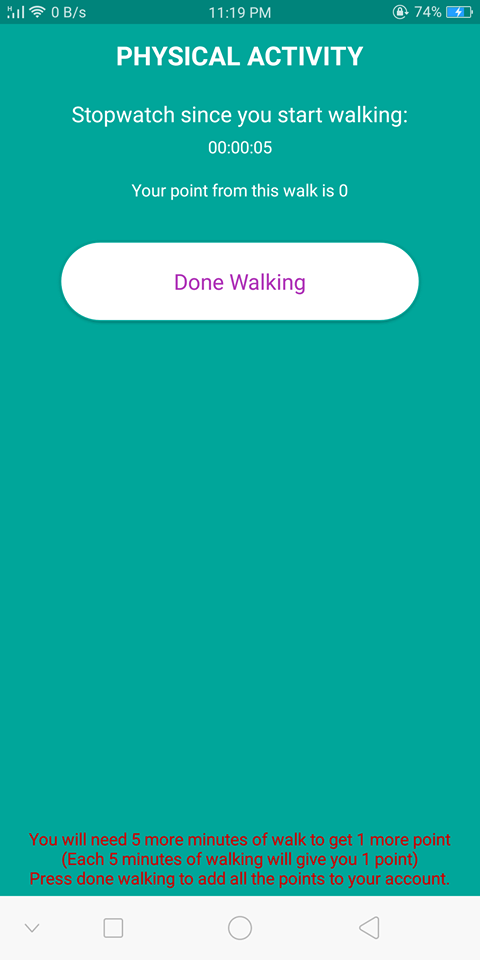


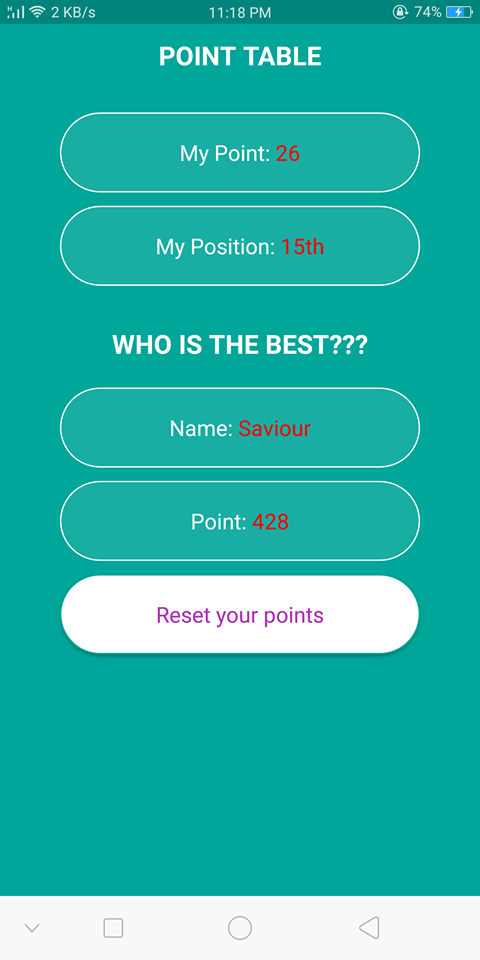


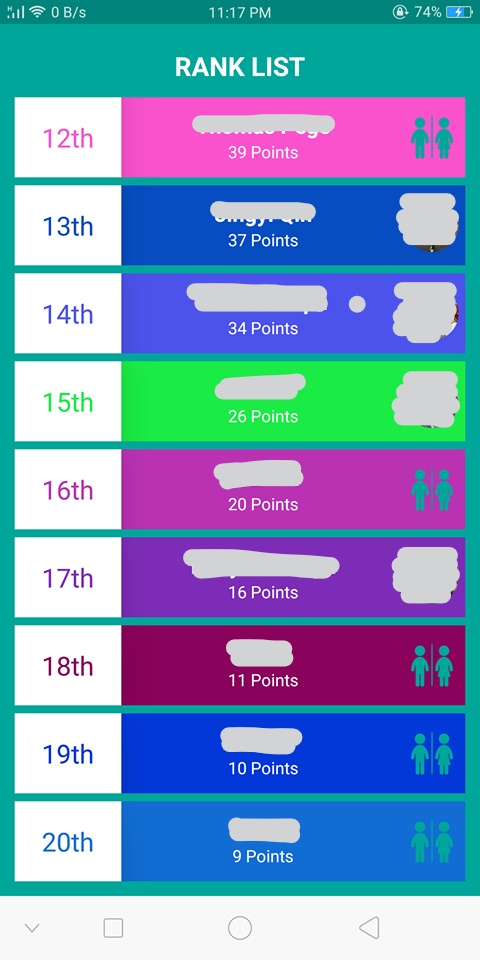


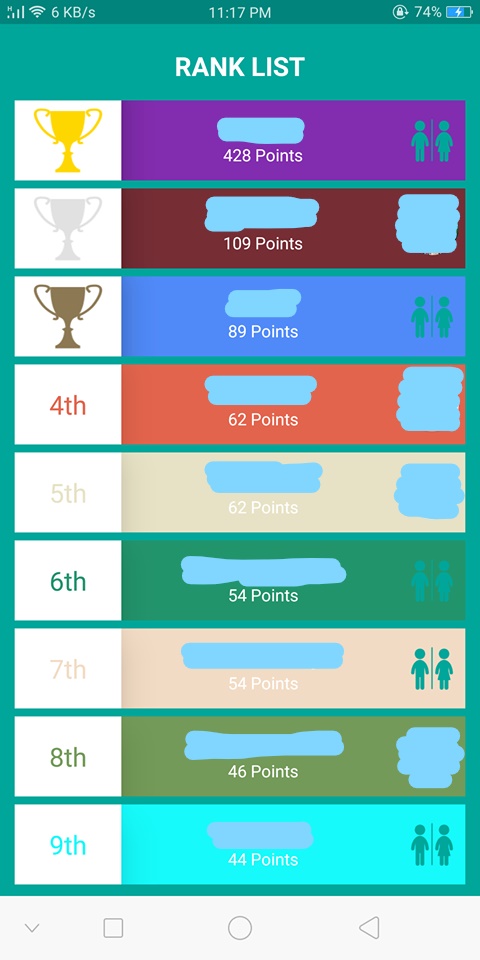

Supplement: Multimedia Appendix 1 [file formative_v4i5e15083_app1.docx]
