# Supplementary material for: A Persuasive mHealth Behavioral Change Intervention for Promoting Physical Activity in the Workplace: Feasibility Randomized Controlled Trial
Source: JMIR Form Res. 2020 May 4;4(5):e15083. doi: 10.2196/15083 (PMC7235808; doi:10.2196/15083)
Supplement: Multimedia Appendix 2 [file formative_v4i5e15083_app2.docx]

Researcher: Sanaul Haque; MSc

Department: Department of Medical Technology, University of Oulu, Finland

Telephone number: +8801756745776/+358469557272/+3553892096467

**INFORMED CONSENT FOR PARTICIPATION IN RESEARCH ACTIVITIES**

**Project Title: Persuasive Health and Well-being Application in Promoting Physical Activity**

1. INTRODUCTION/PURPOSE:

You are being asked to participate in a research study. The purpose of this study is to promote physical activity among people at the office environment using iGO persuasive application (a prototype of an app). The main objectives of the research study are to explore the effect of persuasive applications on a sense of assess and measurement of a healthier lifestyle in the context of individuals’ productivity (e.g. employees’ performance). About 130 individuals will be invited to participate. The participants/test person should be the healthy adult person who wants to increase their physical activity at the office environment.

1. PROCEDURES:

Test person is asked to participate in using a health application in a smartphone for doing physical activity (e.g., walking/exercise). Test persons will use the app, and their responses will be gathered from forms built into the app (data will be collected to the data server). Data of the participants will be collected from the server of the app (admin can retrieve the data)

Participants will be carried out the study for four weeks. Test person will use the app in a smartphone each of the weekdays for two times, i.e., one session after the breakfast and one session after lunch. Each session will last for 10 minutes. In addition, test persons can also use the app after finishing the two sessions. Basic information as height, weight, age, gender and disability condition are asked. Test persons will be provided a pre-questionnaire and post-questionnaire before and after using the app. A face-to-face interview will also be conducted after the test period. The study will be carried out for four weeks, but participants are free to withdraw/stop using the app at any time. Test person can use the app for testing by downloading it from the playstore (the app is compatible with android based smartphones only). Test persons can borrow either an android smartphone from researcher-Sanaul Haque or he/she can use his/her own smartphone. But the borrowed smartphone must be returned to the researcher after the test study.

1. RISKS AND BENEFITS:

Participation in this study does not involve any significant risks. The result of the study will benefit the research and development of technologies for supporting wellness.

1. CONFIDENTIALITY:

Any information learned and collected from this study will remain confidential. The researcher (s) will keep my personal information confidential and the results are published so that the test person is not identifiable. To help protect my confidentiality, data are stored at filing cabinets, password-protected computers, and identification codes instead of person names are used as identification of data. Only the members of the research team will have access to these records.

Consenting to participate in this research also indicates my agreement that all information collected may be used by current and future researchers in such a fashion that my personal identity will be protected. Such use will include sharing anonymous information with other researchers for checking the accuracy of study findings and for future approved research that has the potential for improving human knowledge.

1. SPONSOR OF THE RESEARCH:

This study is partly funded by UNIOGS Graduate School, University of Oulu, Finland

1. COMPENSATION/COSTS:

Participation in this study will involve no cost to test person and test persons are not paid for participation.

CONTACTS AND QUESTIONS:

The researcher, Sanaul Haque, Sanaul Haque (md.haque@oulu.fi),

Other research persons

Prof. Timo Jämsä ([timo.jamsa@oulu](mailto:timo.jamsa@oulu)), supervisor for the research study

Ph.D. Maarit Kangas ([maarit.kangas@oulu.fi](mailto:maarit.kangas@oulu.fi)), supervisor for the Ph.D. study

1. VOLUNTARY PARTICIPATION

I have been informed that my participation in this research study is voluntary and that I am free to withdraw or discontinue participation at any time. I have been informed that data collected for this study will be retained by the researcher and analyzed even if I choose to withdraw from the research. If I do choose to withdraw, the researchers may use my data up to the time I decide to withdraw.

***I will be given a copy of this consent form to keep.***

1. SIGNATURE FOR CONSENT

The above-named researcher has answered my questions and I agree to be a research participant in this study.

I agree

I do not agree

I have borrowed a smartphone for testing the app.

I give permission to keep the record of my information in using the health app

I do not give permission to keep the record of my information in using the health app my answers regarding the research topic and record my voice or image and use in scientific publications or presentations.

Participant’s Name: ________________________________ Date: ______________________

Participant’s Signature: _____________________________ Date: ______________________

Researcher's Signature: _____________________________ Date: __________________________

Researcher: Sanaul Haque; MSc

Department: Department of Medical Technology, University of Oulu, Finland

Telephone number: +8801756745776/+358469557272/+3553892096467

**INFORMED CONSENT FOR PARTICIPATION IN RESEARCH ACTIVITIES**

**Project Title: Persuasive Health and Well-being Application in Promoting Physical Activity**

1. INTRODUCTION/PURPOSE:

You are being asked to participate in a research study. The purpose of this study is to promote physical activity among people in the office environment using a paper diary. The main objectives of the research study are to explore the effect of mHealth and persuasive applications on a sense of assess and measurement of a healthier lifestyle in the context of individuals’ productivity (e.g., employees’ performance). About 100 individuals will be invited to participate. The participants/test person should be the healthy adult person who wants to increase their physical activity at the office environment.

1. PROCEDURES:

The test person is asked to participate in using a paper diary for doing physical activity (e.g., walking/exercise). Test persons will use the paper diary, and their responses will be gathered from the filled diary form (only research admin can retrieve the data and it will be used for the research purpose only such as writing conference and journal article etc.)

Participants will be carried out the study for four weeks. Test person will use the paper instructions each of the weekdays for two times i.e. one session after the breakfast and one session after the lunch. Each session will last for 10 minutes. Basic information as height, weight, age, gender and disability condition are asked. Test persons will be provided a questionnaire after using the paper diary. A face-to-face interview will also be conducted after the test period. The study will be carried out for a month trial period, but participants are free to withdraw using the paper diary at any time.

1. RISKS AND BENEFITS:

Participation in this study does not involve any significant risks. The result of the study will benefit the research and development of technologies for supporting wellness.

1. CONFIDENTIALITY:

Any information learned and collected from this study will remain confidential. The researcher (s) will keep my personal information confidential and the results are published so that test person is not identifiable. To help protect my confidentiality, data are stored at filing cabinets, password-protected computers, and identification codes instead of personal names are used as identification of data. Only the members of the research team will have access to these records.

Consenting to participate in this research also indicates my agreement that all information collected may be used by current and future researchers in such a fashion that my personal identity will be protected. Such use will include sharing anonymous information with other researchers for checking the accuracy of study findings and for future approved research that has the potential for improving human knowledge.

1. SPONSOR OF THE RESEARCH:

This study is partly funded by UNIOGS Graduate School, University of Oulu, Finland

1. COMPENSATION/COSTS:

Participation in this study will involve no cost to test person and test persons are not paid for participation.

CONTACTS AND QUESTIONS:

The researcher, Sanaul Haque, (md.haque@oulu.fi),

(tel. +8801756745776/+358469557272/+353872096467)

Other research persons

Prof. Timo Jämsä ([timo.jamsa@oulu](mailto:timo.jamsa@oulu)), supervisor for the research study

Maarit Kangas ([maarit.kangas@oulu.fi](mailto:maarit.kangas@oulu.fi)), supervisor for the research study

1. VOLUNTARY PARTICIPATION

I have been informed that my participation in this research study is voluntary and that I am free to withdraw or discontinue participation at any time. I have been informed that data collected for this study will be retained by the researcher and analyzed even if I choose to withdraw from the research. If I do choose to withdraw, the researchers may use my data up to the time I decide to withdraw.

***I will be given a copy of this consent form to keep.***

1. SIGNATURE FOR CONSENT

The above-named researcher has answered my questions and I agree to be a research participant in this study.

I agree

I do not agree

I have borrowed a smartphone for testing the app.

I give permission to keep the record of my information in using the health app

I do not give permission to keep the record of my information in using the health app my answers regarding the research topic and record my voice or image and use in scientific publications or presentations.

Participant’s Name: ________________________________ Date: ______________________

Participant’s Signature: _____________________________ Date: ______________________

Researcher's Signature: _____________________________ Date: __________________________
