# Supplementary material for: A Persuasive mHealth Behavioral Change Intervention for Promoting Physical Activity in the Workplace: Feasibility Randomized Controlled Trial
Source: JMIR Form Res. 2020 May 4;4(5):e15083. doi: 10.2196/15083 (PMC7235808; doi:10.2196/15083)
Supplement: Multimedia Appendix 3 [file formative_v4i5e15083_app3.docx]

**Increase your Physical Activity at office**

Attach the instructions sheet on your desk or keep it with you. Walking after the breakfast and lunch will allow you to increase your productivity and burn your calorie and fat from body.

**Walking after Breakfast (00.00-00.00 am)**

If you have your breakfast, please walk for 10 minutes, you can walk alone or with your colleagues.

**Walking after Lunch (00.00-00.00 pm)**

If you have your lunch, please walk for 10 minutes, you can walk alone or with your colleagues.

**Tracking your record:**

Please note down in a personalised calendar your everyday walking data e.g. how many minutes you walk every day or even if you don’t walk.

Breakfast record

| Time | Day 1 | Day 2 | Day 2 | Day 4 | Day 5 |
| --- | --- | --- | --- | --- | --- |
| Week 1 |  |  |  |  |  |
| Week 2 |  |  |  |  |  |
| Week 3 |  |  |  |  |  |
| Week 4 |  |  |  |  |  |

Lunch Record

| Time | Day 1 | Day 2 | Day 2 | Day 4 | Day 5 |
| --- | --- | --- | --- | --- | --- |
| Week 1 |  |  |  |  |  |
| Week 2 |  |  |  |  |  |
| Week 3 |  |  |  |  |  |
| Week 4 |  |  |  |  |  |
