# Supplementary material for: A Persuasive mHealth Behavioral Change Intervention for Promoting Physical Activity in the Workplace: Feasibility Randomized Controlled Trial
Source: JMIR Form Res. 2020 May 4;4(5):e15083. doi: 10.2196/15083 (PMC7235808; doi:10.2196/15083)
Supplement: Multimedia Appendix 4 [file formative_v4i5e15083_app4.docx]

Appendix 4. Quantitative questionnaire on the participants’ experience regarding their physical activity increasing, and three SDT needs of autonomy, competence, and relatedness.

| Increasing physical activity   - Experimental Group: “How much do you agree with this statement: During the month I used the application I had a very strong motivation for physical activity for walking or other activities?” - Control Group: “How much do you agree with this statement: During the month I used the paper instructions I had a very strong motivation for physical activity for walking or other activities?”   Autonomy   - Experimental Group: “How much do you agree with this statement: During the month I used the application I had a very strong motivation for “Physical activity alone” e.g., walking alone?” - Control Group: “How much do you agree with this statement: During the month I used the paper instructions I had a very strong motivation for “Physical activity alone” e.g., walking alone?”   Competence   - Experimental Group: “During the month you used the application how did you find it to see the physical activity display (rank list/leader board) in a social environment in our app with social connection display?” - Control Group: “During the month you used the application how did you find it to see the physical activity display (rank list/leader board) in a social environment in our app with social connection display?   Relatedness   - Experimental Group: “How much do you agree with this statement: During the month I used the application I had a very strong motivation for “Physical activity with others” e.g., walking with others?” - Control Group: “How much do you agree with this statement: During the month I used the paper instructions I had a very strong motivation for “Physical activity with others” e.g., walking with others? |
| --- |

Qualitative questionnaire on the participants’ experience regarding their use experience of the iGO and paper diary.

| For questions in below, please answer these open-ended questions as you see fit:  Question 1   - Experimental Group: “In what ways (if any) did this iGO app help you improve your physical activity? Were there any personal or creative ways you used this app to help with your physical activity?” - Control Group: “In what ways (if any) did this paper diary help you improve your physical activity? Were there any personal or creative ways you used this app to help with your physical activity”   Question 2   - Experimental Group: “Were there any ways in which this app did NOT help you, or made your physical activity worse?” - Control Group: “Were there any ways in which this paper diary did NOT help you, or made your physical activity worse?”   Question 3   - Experimental Group: “In what ways, do you think this app could be improved?” - Control Group: “In what ways, do you think this paper diary could be improved?”   Question 4   - Experimental Group: “What additional features would you like to see in this app?” - Control Group: “What additional features would you like to see in this paper diary?   Question 5   - Experimental Group: “Any additional comments/feedback please?” - Control Group: “Any additional comments/feedback please?” |
| --- |
